# Biallelic *TET2* mutations and canonical *ASXL1* mutations are frequent and cooccur in Blastic Plasmacytoid Dendritic Cell Neoplasm (BPDCN): An institutional experience and review of literature

**DOI:** 10.1002/jha2.617

**Published:** 2023-01-04

**Authors:** Xi Zhang, Eric D. Hsi, Genevieve M. Crane, Yu‐Wei Cheng

**Affiliations:** ^1^ Department of Laboratory Medicine and Pathology Mayo Clinic Rochester Minnesota; ^2^ Department of Pathology Wake Forest University School of Medicine Winston‐Salem North Carolina; ^3^ Department of Laboratory Medicine Cleveland Clinic Cleveland Ohio

**Keywords:** acute leukemia, dendritic cells, molecular genetics

## Abstract

Blastic plasmacytoid dendritic cell neoplasm (BPDCN) is recurrently mutated in epigenetic pathway genes. We studied the myeloid‐related genetic mutations in a cohort of five patients with BPDCN and one paired relapse case at our institution and identified a high frequency of biallelic *TET2* and canonical *ASXL1* (c.1934dupG) mutations. The number of cases is small, but the variant allele fraction (VAF) sums of the *TET2* mutations, as well as the persistence of *TET2* mutations in a case of relapsed BPDCN, suggest an ancestral/founder nature of TET2 clones in the cases. Further literature review shows a high frequency of biallelic *TET2* mutations in reported cases of BPDCN.

1

Blastic plasmacytoid dendritic cell neoplasm (BPDCN) is a rare and aggressive type of hematologic malignancy derived from plasmacytoid dendritic cell precursor cells. Next‐generation sequencing (NGS) techniques have expanded the understanding of the genetics of BPDCN. Epigenetic pathway genes (*ASXL1, TET2, IDH2, EZH2, DNMT3A, ARID1A*, etc.) as well as *TP53* are frequently mutated [1]. About half of the cases harbored at least 1 mutated epigenetic pathway gene. Targeted epigenetic‐based therapies have been proposed as a promising therapeutic approach to BPDCN. In addition, mutations in a number of other genes, including RAS family genes (*KRAS, NRAS*, etc.), IKAROS family genes (*IKZF1, IKZF2, IKZF3*, etc.), *ABL1, JAK2, FLT3*, and *NPM1* have also been reported in a subset of BPDCN cases. Our study was aimed to identify genetic mutations in our BPDCN cohort, and compare our findings to published BPDCN cohorts. Our work identified a high frequency of biallelic *TET2* mutations and canonical *ASXL1* mutations with cooccurrence in a subset of cases.

BPDCN cases were identified from the pathology archives at our institution with 6 cases from 5 patients having sufficient material for further evaluation. This study was approved by the Institutional Review Board. Most of the cases were positive for CD4, CD56, CD123, and TCL1, while negative for relevant lineage specific markers, including CD3, CD19, CD20, and myeloperoxidase (Table 1). All cases underwent expert hematopathologic review as part of subspecialized practice routine at our center. Clinical and laboratory data were obtained by review of electronic medical records. Case 5 (initial skin lesion) and case 6 (relapse in bone marrow after 1 year) were from the same patient (Table 1). Two patients had histories of other myeloid neoplasms in addition to BPDCN, including myelodysplastic syndrome (case 2) and chronic myelomonocytic leukemia (case 3). Eighty percent (4/5) of the patients had bone marrow involvement by BPDCN. Conventional cytogenetic analysis was performed on metaphase cells prepared from bone marrow aspirates using standard techniques and the results were reported using the International System for Human Cytogenetic Nomenclature, 2016 [2]. Both cases (1 and 3) with bone marrow involvement showed complex karyotypes with multiple chromosomal abnormalities (Table 1).

**TABLE 1:** Clinical and pathological features of the BPDCN cohort

Patient no.	Case no.	Other myeloid or lymphoid diseases	Sites of lesion	Positive markers	Negative markers	Specimen tissue source	Blast count (%)	Cytogenetics
1	1	None	Multiple erythematous rashes over chest, upper abdomen, back, and neck, and marrow involvement	CD2, CD7, CD56, CD123, HLA‐DR	Myeloperoxidase, CD19, CD20, CD3	Bone marrow	54	44,XY,‐9,t(12;22)(p11.2;p11.2),‐13,add(15)(p11.2),add(17)(p11.2)[9]/43,idem,add(10)(p13),‐20[cp7]/46,XY[4]
2	2	MDS, MBL, LGL	Multiple tumors involving neck, upper back and flank, and marrow involvement	CD123, CD2, TCL‐1 (strong), CD4 (weak)	CD34, CD117, CD56, CD3, CD8	Bone marrow	40	Not performed
3	3	CMML‐1	Diffuse hyperpigmented and erythematous lesions, few petechiae, coin‐sized bruises over legs and abdomen, and marrow involvement	CD4, CD7, CD13 (variable), CD33, CD38, CD45, CD56, CD117 (variable), CD123, TCL‐1, HLADR	Myeloperoxidase, CD2, CD117	Bone marrow	90	45,XY,del(2)(p13),+8,del(9)(p13),‐9,‐13,add(22)(p11.2)[3]/46,XY[cp2]
4	4	None	Left flank and left buttock erythematous lesions, no bone marrow involvement	CD43, CD45, CD4, CD56, CD123	CD3, CD20, CD5, CD30, CD34, CD117, myeloperoxidase, muramidase, CD33, PAX5	Skin	N/A	46,XY[20] (bone marrow not involved)
5	5	None	Multiple skin lesions involving scalp, forehead, scapula, and upper back	CD4, CD56, CD2, CD123	CD3, CD5, CD8, CD13, CD33, CD117, CD114, CD34	Skin	N/A	Not performed
5	6	None	Relapse in bone marrow	CD4, CD56, CD123	Myeloperixidase, CD3, CD20, CD34, CD117	Bone marrow	Single aggregate	Not performed

Genomic DNA extracted from involved bone marrow or skin biopsies was amplified and subjected to mutation analysis. Cases 1 and 2 were sequenced using the Oncomine Myeloid Research Assay of 40 genes and analyzed with Thermo Fisher Scientific Ion Reporter (Waltham, MA). Cases 3 through 6 were sequenced using the institutional Myeloid Neoplasm Next‐Generation Sequencing panel of 53 genes on Illumina platform (San Diego, CA). All variants identified were in genes included in both panels. The limit of detection for the reported variants was set at 5% VAF. Among the newly diagnosed BPDCN cases [1, 2, 3, 4, 5], gene level mutations were detected in all patients. The average number of mutations per case was 5.2 in the new BPDCN cases. The epigenetic modifier genes were the most frequently mutated genes, in line with previous findings (Figure 1A). In this group of genes, the most common mutations occurred in *TET2* and *ASXL1*, seen in 4 (80%) and 3 (60%) patients, respectively. Two (40%) patients had both *TET2* and *ASXL1* mutations. *TET2* and *IDH2* mutations were mutually exclusive, a pattern consistent with other myeloid neoplasms.

**FIGURE 1:**
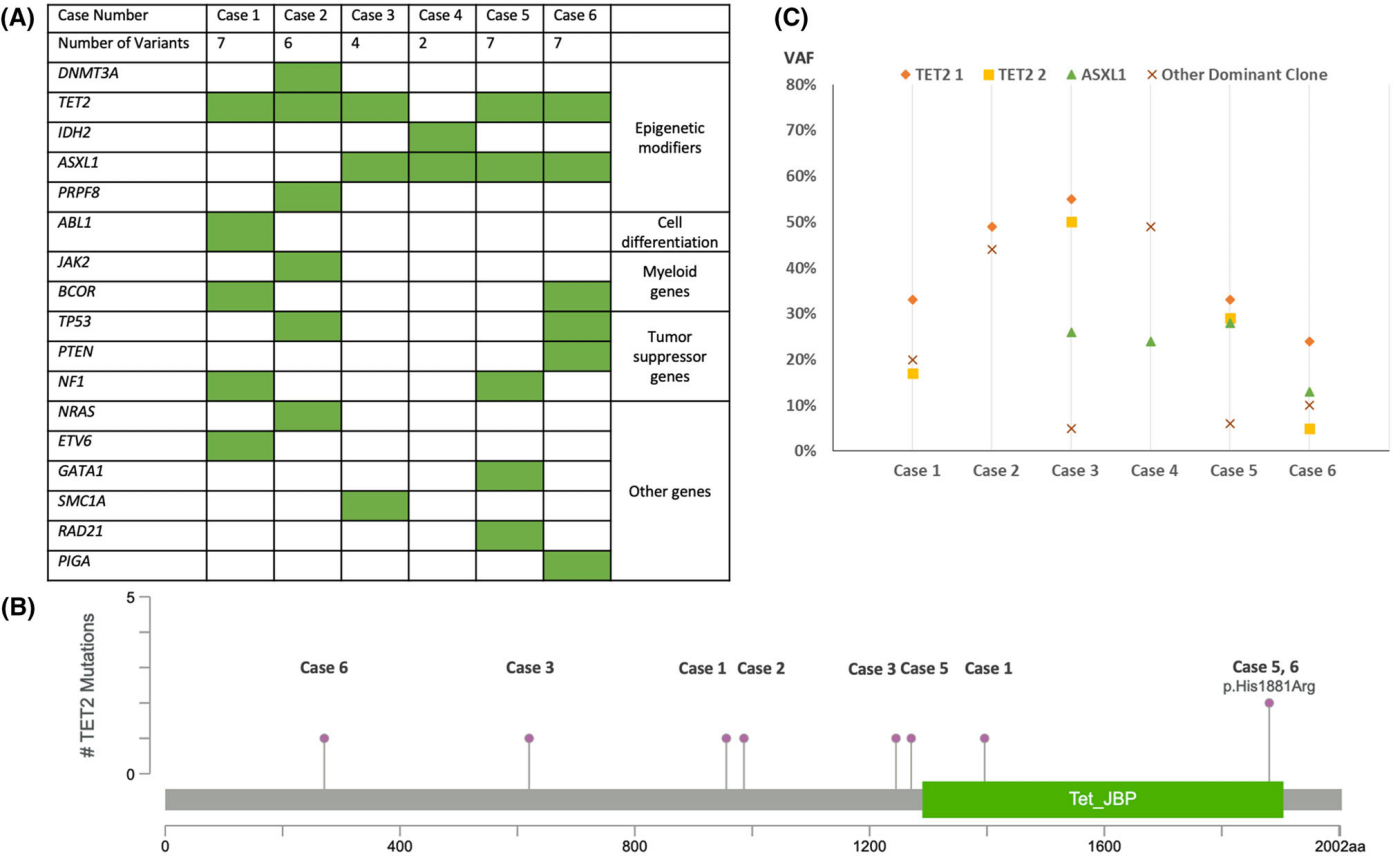
(A) List of gene mutations in the BPDCN cohort. (B) The locations of the *TET2* mutations in the BPDCN cases. (C) The VAFs of the dominant (TET2 1) and secondary (TET2 2) *TET2* clones, *ASXL1* clone and other dominant clones in the BPDCN cases

Interestingly, our cohort showed frequent biallelic *TET2* mutations spread across the coding exons of the gene (3/5 cases at diagnosis, Figure 1B). Mutations in *TET2* included 1 frameshift mutation, 5 missense mutations, and 1 nonsense mutation, with the VAFs ranging from 17% to 55%. The majority of *TET2* mutations were non‐recurrent. In the cases with more than one *TET2* mutation, the VAF sums of the two mutations were all greater than or equal to 50%. The VAFs of the *TET2* mutations were also greater than most other gene mutations, suggesting the likely founder/ancestral nature of *TET2* mutated clones (Figure 1C). Patient 5 was initially diagnosed with BPDCN in a skin lesion, and subsequently treated with CHOP chemotherapy followed by consolidative autologous bone marrow transplant. Unfortunately, he developed a cutaneous lesion over the forehead, which was biopsied and found to be consistent with recurrence of disease. Bone marrow was also involved by disease at the time of recurrence. A previously identified *TET2* mutation was present at time of relapse, together with a few additional mutations in other genes, suggesting persistent molecular disease and clonal progression.

*TET2* encodes a dioxygenase that converts 5‐methylcytosine (5‐mC) to 5‐hydroxymethylcytosine (5‐hmC) and promotes DNA demethylation. *TET2* mutations are generally loss of function variants (frameshift and nonsense) that could be monoallelic or biallelic and occur throughout the length of the gene. Biallelic *TET2* mutations have been reported in 10%–30% of MDS and AML patients and approximately 30% of CMML patients [3, 4]. The Biallelic *TET2* mutations were founder lesions in 72% of CMML cases in one study [3]. Biallelic *TET2* mutations likely result in a relatively more competitive advantage over single *TET2* mutations [5]. Moreover, the *TET2* mutant allele dosage has potential theranostic impact for myeloid neoplasms. MDS patients with 1 or more *TET2* mutations showed response to hypomethylating agents such as azacitidine (AZA) in some studies [6]. Higher abundance *TET2* mutations is associated with increased response to hypomethylating agents [7].

BPDCN could be accompanied by other myeloid malignancies. Recent studies showed that some cases of concomitant BPDCN and CMML shared the same clonal origin [8, 9]. *TET2* loss‐of‐function was a shared ancestral genetic event in some reported cases before divergent clonal evolution occurred in each neoplasm [10]. We did a literature review of previous published BPDCN cohorts and investigated the frequency of biallelic *TET2* mutations. The type and frequency of *TET2* mutations in BPDCN appear similar to those observed in other myeloid neoplasms [11, 12]. However, the frequency of biallelic *TET2* mutations appears significantly higher in BPDCN compared to other myeloid malignancies (Supplemental Table S1). The fraction of cases with biallelic *TET2* mutations is likely underestimated, since comprehensive *TET2* mutational profiling ideally incorporates sequencing analysis as well as copy number analysis to identify complex genomic alterations affecting the *TET2* locus.

All 3 *ASXL1* mutations were the c.1934dupG (p.Gly646Trpfs*12) variant, with the VAFs ranging from 24% to 28%. *ASXL1* encodes a polycomb repressive complex protein with a vital role in chromatin regulation. *ASXL1* mutations are commonly frameshift or nonsense, and most are located within or upstream of the catalytic domain, which causes C‐terminal truncation of the protein. The *ASXL1* G646fs*12 mutation is a hotspot mutation and commonly referred to as the “canonical” *ASXL1* mutation. The *ASXL1* G646fs*12 mutation accounts for approximately half of all *ASXL1* mutations in AML [13] and approximately 39% of all *ASXL1* mutations in reported BPDCN cases (Supplemental Table S1). Although the potential interaction between *ASXL1* and *TET2* mutations could not be reliably inferred from the mutation frequencies of a small number of cases, it is interesting to note that in cases 3 and 5 in which *TET2* and *ASXL1* mutations coexisted, the VAFs of the mutations were much higher than other clones, suggesting that the *TET2* and *ASXL1* mutations cooccurred in the same dominant clones, with the *ASXL1* mutation acquired at a later time point than the *TET2* mutation (Figure 1C).

We identified, for the first time, pathogenic or likely pathogenic mutations in *SMC1A* and *PRPF8* genes in BPDCN. *SMC1A* gene encodes a subunit of the cohesin complex which mediates sister chromatid cohesion and homologous recombination [14]. *PRPF8* gene encodes a spliceosome component essential in pre‐mRNA splicing and is frequently mutated in CMML [15]. The VAFs of the *SMC1A* and *PRPF8* mutations suggest that they were subclonal genetic alterations in the respective cases.

In summary, we report high frequencies and cooccurrence of biallelic *TET2* mutations and canonical *ASXL1* mutation in a BPDCN cohort and literature. Mutations in epigenetic modifier genes play an early role in the pathogenesis of BPDCN and represent a poor prognostic factor. As molecular genetic findings of this rare disease accumulate, further investigation is needed to elucidate the clonal evolution involving *TET2* and *ASXL1* mutations, as well as their prognostic and theranostic values in this entity.

## AUTHOR CONTRIBUTIONS

X.Z. interpreted the sequencing data and wrote the manuscript. E.H., G.M.C., and Y.C. reviewed the manuscript. The study was supervised by all the authors. The drafting and revision of the manuscript were done by all authors. All authors have read and agreed to the published version of the manuscript.

## FUNDING

The authors received no specific funding for this work.

## CONFLICT OF INTEREST

The authors declare they have no conflicts of interest.

## ETHICS STATEMENT

This study was performed following IRB approval (Cleveland Clinic Foundation, Study #21‐643, entitled “Next Generation Sequencing Analysis of Rare Types of Myeloid Neoplasms”). This study also underwent review in the Case Comprehensive Cancer Center Protocol Review and Monitoring Committee (PRMC) due to the inclusion of cancer patients, to further ensure ethical retrospective review of medical records. All study personnel were certified as having up to date relevant training in research ethics, compliance and safety.

## PATIENT CONSENT STATEMENT

Following detailed review of the study, the requirement for patient consent was waived by the IRB due to the minimal risk to the subjects and historical nature of the data.

## Supporting information

Supplemental Information

## Data Availability

The data that support the findings of this study are available from the corresponding author upon reasonable request.
