# Supplementary material for: Biallelic TET2 mutations and canonical ASXL1 mutations are frequent and cooccur in Blastic Plasmacytoid Dendritic Cell Neoplasm (BPDCN): An institutional experience and review of literature
Source: EJHaem. 2023 Jan 4;4(1):236–40. doi: 10.1002/jha2.617 (PMC9928664; doi:10.1002/jha2.617)
Supplement: Supplementary file 1 — Supplemental Information [file JHA2-4-236-s001.docx]

Supplemental Table 1. Frequencies of *TET2* and *ASXL1* Mutations in Published BPDCN Cohorts

| Study | Cohort size | Mono-allelic *TET2* mutation | Biallelic/multi-allelic *TET2* mutation/inactivation | Canonical *ASXL1* mutation | Other *ASXL1* mutation |
| --- | --- | --- | --- | --- | --- |
| Our study | 6 | 1 | 3 | 3 | 0 |
| Menezes, et al, 2014 | 25 | 8 | 1 | 0 | 8 |
| Sapienza, et al, 2019 | 14 | 2 | 1 | 0 | 4 |
| Renosi, et al, 2020 | 13 | 0 | 8 | 3 | 3 |
| Yin, et al, 2021 | 50 | 14 | 14 | 11 | 12 |
| Jardin, et al, 2011 | 13 | 4 | 3 | N/A | N/A |
| Alayed, et al, 2013 | 5 | 2 | 2 | N/A | N/A |
| Total | 126 | 31 | 32 | 17 | 27 |
